# Quick Fabrication of Large-area Organic Semiconductor Single Crystal Arrays with a Rapid Annealing Self-Solution-Shearing Method

**DOI:** 10.1038/srep13195

**Published:** 2015-08-18

**Authors:** Yunze Li, Deyang Ji, Jie Liu, Yifan Yao, Xiaolong Fu, Weigang Zhu, Chunhui Xu, Huanli Dong, Jingze Li, Wenping Hu

**Affiliations:** 1 State Key Laboratory of Electronic Thin Films and Integrated Devices, School of Microelectronics and Solid-State Electronics, University of Electronic Science and Technology of China, Chengdu 610054, China; 2 Beijing National Laboratory for Molecular Sciences, Key Laboratory of Organic Solids, Institute of Chemistry, Chinese Academy of Sciences, Beijing 100190, China

## Abstract

In this paper, we developed a new method to produce large-area single crystal arrays by using the organic semiconductor 9, 10-bis (phenylethynyl) anthracene (BPEA). This method involves an easy operation, is efficient, meets the demands of being low-cost and is independent of the substrate for large-area arrays fabrication. Based on these single crystal arrays, the organic field effect transistors exhibit the superior performance with the average mobility extracting from the saturation region of 0.2 cm^2^ V^−1^s^−1^ (the highest 0.47 cm^2^ V^−1^s^−1^) and on/off ratio exceeding 10^5^. In addition, our single crystal arrays also show a very high photoswitch performance with an on/off current ratio up to 4.1 × 10^5^, which is one of the highest values reported for organic materials. It is believed that this method provides a new way to fabricate single crystal arrays and has the potential for application to large area organic electronics.

Organic single crystals are the best form to reveal intrinsic properties of the bulk materials and to understand structure-function relationship due to their perfectly ordered molecules, the absence of grain boundaries and the minimal number of charge traps^1^,^2^,^3^,^4^,^5^,^6^,^7^,^8^,^9^,^10^,^11^,^12^,^13^,^14^. However, it has been acknowledged that organic single crystals have a few weak points, such as random position, crystal size limited in micro/nanometer scale, disordered alignment and low yields^15^,^16^,^17^,^18^,^19^. In addition, the quality of single crystals is largely dependent on the substrate because the slow crystallization process is very sensitive to the environment. For example, silicon dioxide (SiO_2_) is the widely adopted rigid substrate for fabricating organic single crystals, which generally requires to be modified with a self-assembly monolayer such as octadecyltrichlorosilane (OTS) for optimizing the crystallization process^20^,^21^,^22^,^23^,^24^,^25^. Therefore, the practical application of organic single crystals in the microelectronic field is extremely restricted due to the above-mentioned disadvantages. To this end, the development of new methodologies for mass production of one dimensional organic single crystal with high aspect ratio is very critical and meaningful. That is to say, highly-ordered and well-aligned micro/nanowire, or ribbon arrays with the desired orientation, are urgently required and have great potential application for large scale integrated circuits^26^,^27^,^28^,^29^.

Recently, a variety of methods^30^,^31^ have been developed to achieve large-area well-aligned single-crystal arrays such as zone-casting^32^,^33^, dip-coating^34^,^35^,^36^, drop-casting^37^,^38^,^39^and solution-shearing^40^,^41^,^42^. It should be noted that these fabrication processes are time-consuming, which demonstrate these high-quality single crystals are obtained with sacrificing the growth time. Furthermore, the selection of the substrate is unitary, which is mainly limited to silicon dioxide with modified layer. Up to now, the quick creation of large-scale, well-aligned organic single crystals directly on the bare substrate is still a big challenge. 9, 10-bis (phenylethynyl) anthracene (BPEA) is one of the typical organic semiconductors. It has strong fluorescent intensity^43^,^44^ and its single crystal ribbons can be easily prepared using solution-processed method^45^,^46^,^47^,^48^ with the length as long as several hundred micrometers. In this work, we developed a straightforward methodology named as Rapid Annealing Self-Solution-Shearing (RASSS) to quickly produce large-area well-aligned BPEA single crystal arrays. These single crystal arrays with the length reaching up to millimeter scale can be manufactured on different substrates such as Si/SiO_2_, glass and polyimide^49^. Specifically speaking, a slightly tilted substrate is first covered with a thin layer of BPEA solution, and then a high-temperature annealing treatment is applied to make the solvent rapidly volatilize along the direction of the slope. As a consequence, highly ordered single crystal arrays of BPEA are achieved very fast, generally within 10 s. This process can be controlled by the slope degree of the substrate as well as other affecting factors such as annealing temperature and solution concentration. Compared with other technologies^50^,^51^,^52^, it is obvious that our method is time-efficient (a couple of seconds), and easy to operate (without any special instruments or complicated experimental procedures). Meanwhile, the BPEA arrays exhibit good electrical properties with the average mobility of 0.2 cm^2^ V^−1^s^−1^ (the highest 0.47 cm^2^ V^−1^s^−1^) and the ratio of I_ON_/I_OFF_ above 10^6^. In addition, these arrays show a very high photoswitch performance with an on/off current ratio up to 4.1 × 10^5^.

## Results

### The procedure to obtain large-area, highly-ordered single crystal arrays

**Figure 1:**
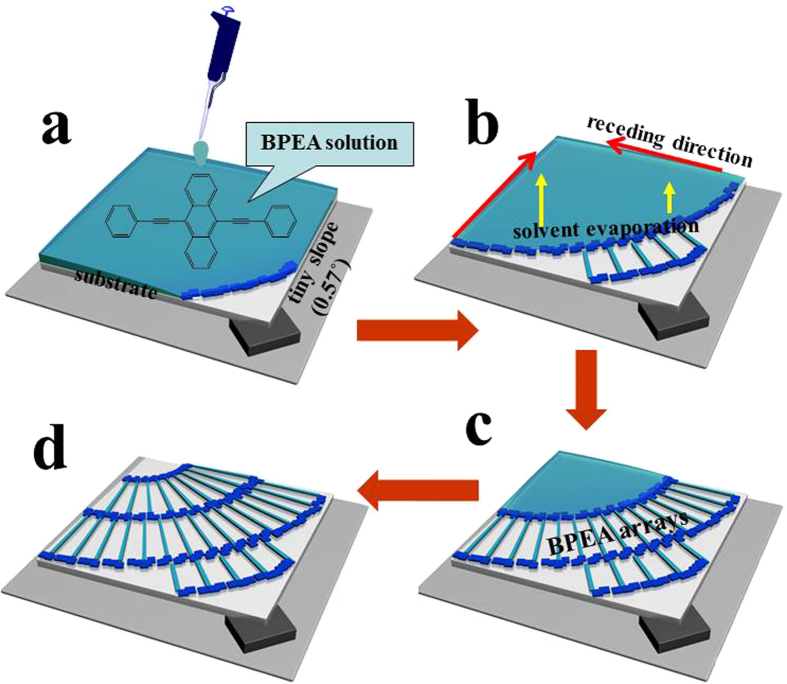
The illustration of experimental procedure to obtain large-area, highly-ordered single crystal arrays. (**a**) A tiny slope is generated by inserting a small piece of coverslip under the corner of the substrate. Then, the setup is kept at a desired temperature for rapid evaporation of the solvent. Finally, the solution is dropped on the substrate directly by a burette until the solution covers the substrate completely (about 70 μL). (**b**,**c**) As the solvent evaporates quickly, the solution recedes along the slope direction rhythmically, resulting in the formation of ordered single crystal arrays. (**d**) The well-aligned single crystal arrays are obtained on the whole substrate surface in the large area.

Figure 1 shows the schematic drawing of the experimental procedure to obtain the single crystal arrays via RASSS method. First, BPEA (the chemical structure shown in the inset of Fig. 1a) powder was dissolved in mesitylene solvent with the concentration ranging from 0.2 mg/ml to 2 mg/ml. Three different substrates (Si/SiO_2_, glass and polyimide) were used. The substrate was placed on the heater which was kept at a high temperature and tilted at a tiny angle (about 0.57°) by inserting a piece of thin coverslip between the surfaces at one corner (Fig. 1a). Then, a burette was brought to drop the BPEA solution (about 70 μL) to cover the whole substrate. The solvent was evaporated quickly from the whole liquid surface, leading to rapidly decreasing the liquid level. At the same time, dense seed crystals were generated at the contact line forming a seed-crystal band. After that, the self-solution-shearing effect guided the growth direction of the single crystal ribbons. Simultaneously, evenly spaced seed crystals grew up following the solution receding along the specific direction (Fig. 1b,c). As a consequence, large-scale and well-aligned ribbon crystals of BPEA were gradually obtained as shown in Fig. 1d. The self-solution-shearing process does not require any complicated technique or expensive equipment, and the whole process can be finished within ten seconds. Finally, the metallic source and drain electrodes were prepared by manually gluing Au-films to construct the bottom-gate top-contact (BGTC) OFETs^53^,^54^. Current-voltage characteristics of the devices were measured in the atmospheric condition using a Keithley 4200-SCS semiconductor parameter analyzer. The morphologies and crystalline structures were characterized by optical microscope, atomic force microscope, transmission electron microscope, and X-ray diffraction technique, respectively.

### Characteristics of the single crystal arrays

**Figure 2:**
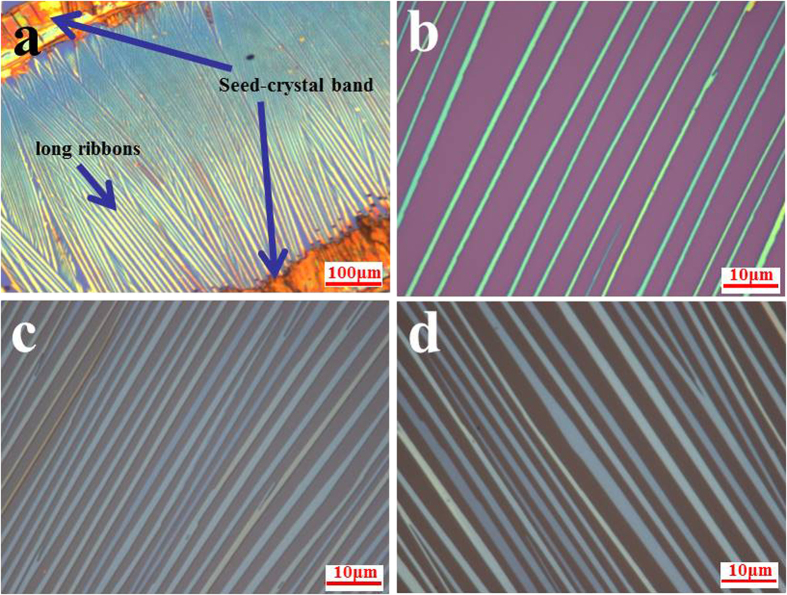
Optical micrographs of BPEA single crystal arrays fabricated on the different substrates. (**a**) Large-area arrays of single crystal ribbons with the length approaching millimeter scale on the SiO_2_ substrate. (**b**) The enlarged image of highly-ordered single crystal arrays on the SiO_2_ substrate, where every individual ribbon has a uniform color and a straight line in the shape. (**c**) Well-aligned organic ribbons achieved on (**c**) PI substrate and (**d**) Glass substrate. These images suggest that the formation of single crystal ribbons is independent of the substrate.

**Figure 3:**
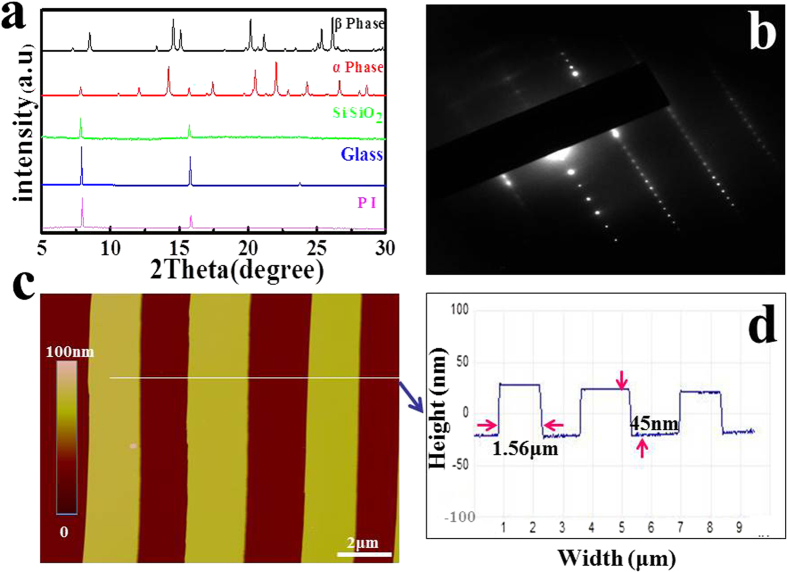
Characterization of the BPEA arrays by different ways. (**a**) XRD patterns of the single crystals generated at the same condition on the different substrates. All patterns have two main peaks at the same location (2θ = 7.8°, 15.7°), which are indexed to α phase of BPEA. (**b**) SAED pattern of a ribbon; the pattern exhibits a single set of parallel spots, indicating the single crystallinity of the ribbons. (**c**) AFM height mode image of the single crystal arrays and the corresponding cross sectional profile, showing the ribbon surface is very smooth (roughness about 0.3 nm), and the ribbon array is uniform with its ribbon width about 1.56 μm and the height about 45 nm.

Using this RASSS method, large-area single crystal arrays with the length at the millimeter scale can be easily obtained, as shown in optical microscope image (Fig. 2a). This phenomenon can be repeated by using different substrate, such as Si/SiO_2_ (Fig. 2b), polyimide (Fig. 2c), and glass (Fig. 2d). From the optical images, it is clear that BPEA on these three substrates is well-aligned with a preferential growth direction along the solution shearing direction. Every individual ribbon had a regular configuration and a uniform color, which indicates that superior quality single crystal is achieved. From the X-ray diffraction patterns shown in Fig. 3a, it is evident that BPEA arrays on different substrates exhibit two main peaks (2θ = 7.8°, 15.7°) and that these two main peaks could be assigned to (200) and (400) of the α phase^46^,^47^. So it is concluded that the BPEA ribbons on different substrates are single crystals. This is also a valuable evidence that the growth of these single crystal arrays has no strong correlation with the used substrate. Besides, the single-crystal arrays fabricated at different temperatures (130 °C, 140 °C, 150 °C) (Fig. S1a) and various concentrations (0.5 mg/ml, 1 mg/ml, 2 mg/ml) (Fig. S1b) were characterized by the X-ray diffraction. The result made the fact clearly that they were all composed of the same α phase of BPEA. The crystal structure of BPEA ribbons was further confirmed by selected area electron diffraction (SAED) (Fig. 3b), which showed a single set of parallel spots indicating the single crystallinity of these ribbons. The surface morphology of individual single crystal ribbon was measured by AFM (height mode mapping). Figure 3c demonstrated a faceted shape with a very smooth surface of averaged roughness about 0.3 nm. And the width of the individual ribbon was about 1 μm and its height was about 45 nm (Fig. 3d).

### Critical parameters of RASSS experiment

During the RASSS process, three critical parameters should be considered. The first one was the angle of the substrate slope, which decided the shearing direction and influenced the shearing speed. The effects of three different angles were tested with other identical experimental conditions (temperature, concentration, location). BPEA crystals grew randomly and disorderly on the horizontal substrate (0°) (Fig. S2a) and became discontinuous spots while the angle was too big (about 1.71° generated by a piece of silicon wafer) (Fig. S2b). Only around the middle state (about 0.57°) was the optimal angle to produce well controlled crystals arrays. The temperature of the heater was another important factor, which had a close relationship with the evaporating rate of the solvent and the shearing speed. Both low temperature (120 °C, Fig. S2c) and high temperature (160 °C, Fig. S2d) could not result in the formation of the single crystal ribbon arrays. BPEA single crystal arrays were produced at a proper temperature (130 °C, 140 °C or 150 °C). The last critical factor is the initial concentration of the solution. The thickness increased about 10 ~ 15 nm with the concentration varying from 0.5 mg/ml (Fig. S3a,c) to 1 mg/ml (Fig. S3b,d) at the same heating temperature. It is noted that when the temperature increased in an allowable scope at the same concentration, the thickness varied within a certain range. This could be explained that higher temperature made the molecule in the solution have higher kinetic energy and when the solution sheared rapidly, more molecules would be transported to construct the single crystals arrays. It was noted that selecting the appropriate solvent was also very important. Besides mesitylene with boiling point about 164 °C, methylbenzene (boiling point, 110 °C) and 1, 2-dichlorobenzene (boiling point, 180 °C) were also adopted into this process. Unfortunately, although the methylbenzene solvent could help to form single-crystal arrays as shown in Fig. S4a, the morphology and the quality was not good as that from mesitylene solution and the performance was relatively low with the mobility of only 2.25 × 10^−3^ cm^2^ V^−1^s^−1^ (Fig. S5). As for high boiling point solvent (1, 2-dichlorobenzene), there was no single crystal obtained (Fig. S4b).

### Controlling the density of single crystal arrays

At the same heating temperature (130 °C, 140 °C or 150 °C), the density increased evidently with the concentration changing from 0.5 mg/ml to 2 mg/ml (Fig. S6). However, at the same concentration but different temperature, the temperature had a slight impact on the density, which indicated that the concentration played an important role to determine the density. All of these could be explained as followed. At the beginning of the growth, a large number of crystal grains were generated at the contact line, but only a few could grow up with the solution shearing rapidly due to the limited resource. However, with the concentration increasing, more crystal grains obtained the chance to grow up for a higher density. So we could control not only the orientation but also the thickness and density of the arrays through our RASSS process.

## Discussions

**Figure 4:**
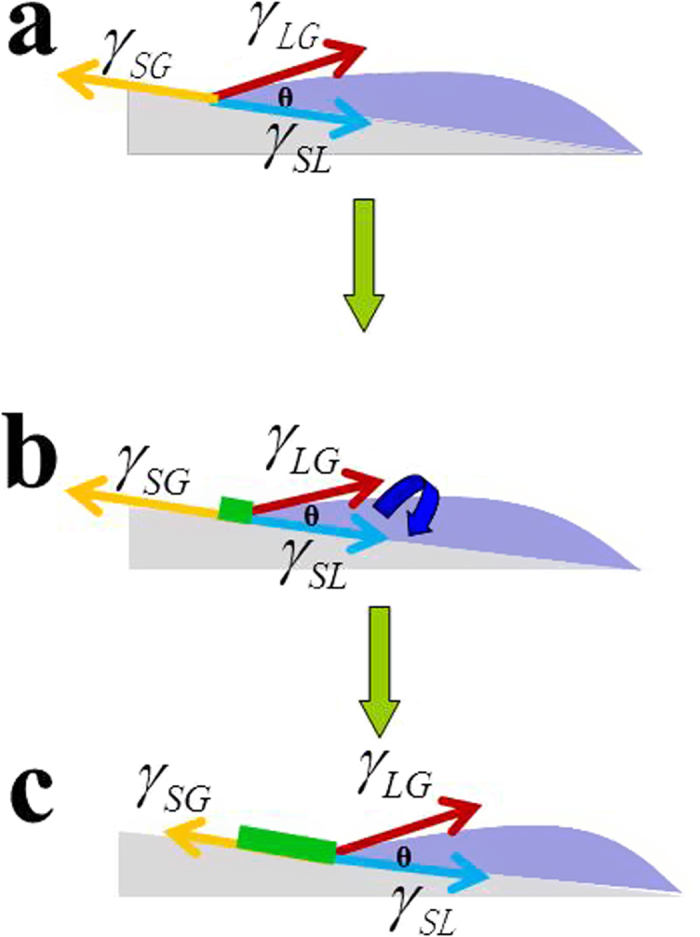
Mechanism of the RASSS method. According to the phenomenon of the experiment, we explain the mechanism depending on the Young equation though this picture. Figure a, b, c show a complete variation at the contact line between the solution and substrate where the interfacial tensions are regarded as the driving force.

The mechanism of our RASSS method can be illustrated in Fig. 4. When a drop of solution spreads over the substrate that had a very small slope, it will reach an equilibrium state quickly with the help of the interfacial tension among the substrate, liquid and gas phase (Fig. 4a). Herein, we think that the effect of gravity can be ignored because of the small slope and microliter scale solution. And the interfacial tension at the interface must satisfy the Young equation which is an basic equation in interfacial science^55^:


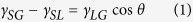


where *γ*_*SG*_ is the interfacial tension between solid and gas phase; 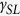 is the interfacial tension between solid and liquid phase; *γ*_*LG*_ is the interfacial tension between liquid and gas phase. All of these parameters are constant except the contact angle θ. When the substrate was placed on the heater (Fig. 4b), the solvent would evaporate quickly because of the thinner solution film. The surface of the solution would gradually be closer to the substrate leading to the decreasing of θ, and then the Young equation was broken following the decreasing of θ. The right side of the equation becomes greater than the left side. According to the mechanics principle, the contact line (for pure solvent) will shear along the direction of the slope to maintain mechanical equilibrium. However, in our experiment, the contact line would be pinned there owing to large amounts of crystal nucleus that generated at the contact line with the evaporating of the solvent. This pinning effect would prevent the interface from receding momentarily. With the solvent evaporation, the local concentration increases and then reaches the saturation state. When the concentration is above the saturation, nucleation occurs to form the contact line. It is noted that higher temperature makes the molecules in solution own higher kinetic energy. This thermal motion brings the molecules to the contact line for the growth of single crystals arrays. The preferential growth direction must have the highest binding force and closely arrangement of molecules. With the θ continuously decreasing, as shown in Fig. 4c, the contact line will shear along the direction of the slope rapidly because of the disappearance of the pinned effect. And the very high receding speed produces a constraining force to guarantee the yield of highly ordered single crystals along the preferential direction. Finally, the system would recover to the initial state and repeat again as illuminated in Fig. S7.

**Figure 5:**
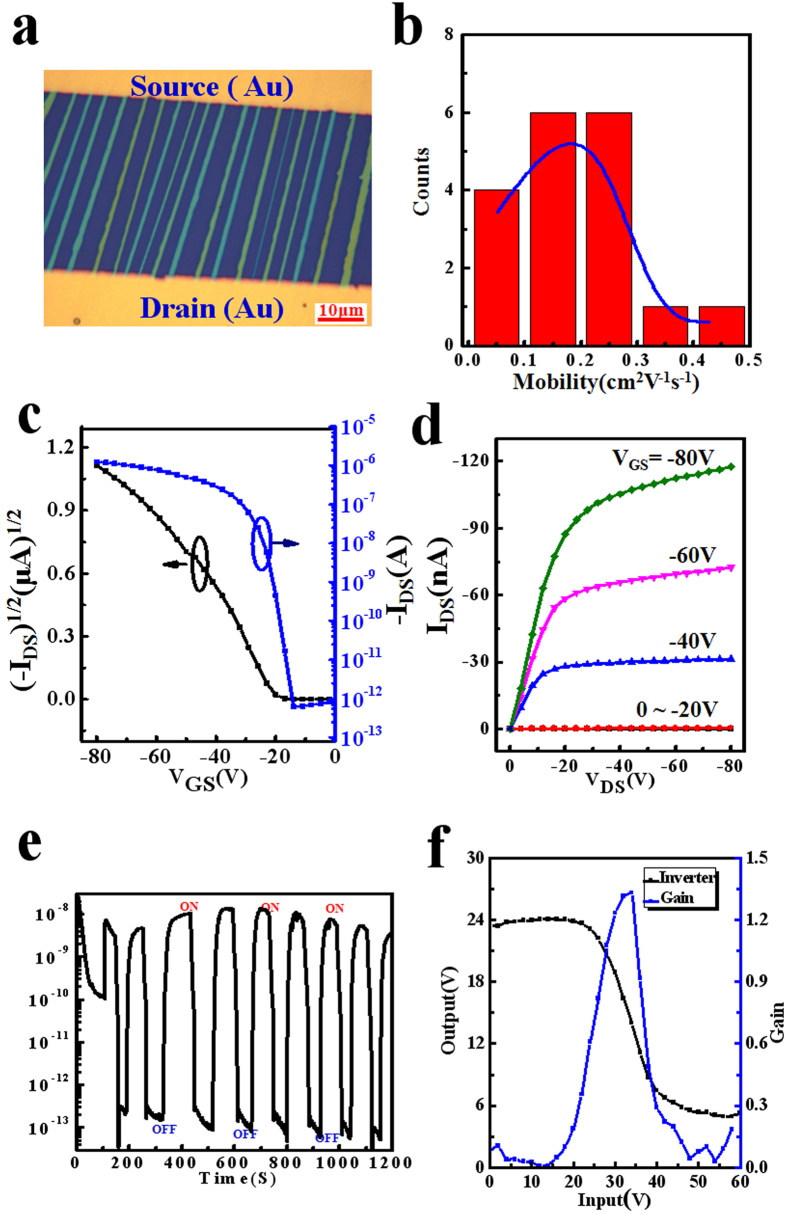
Electrical properties of the BPEA arrays. (**a**) Optical microscope of the FET transistor fabricated by manually gluing Au-films. The active channel width was measured from the contacting area of the crystals which crosses the drain and source electrodes. (**b**) Histograms of field effect mobility, standard deviation: 0.1 and maximum mobility: 0.472 cm^2^V^−1^s^−1^ measured from 18 transistors. (**c**) Transfer (**d**) Output characteristics of a typical device showing a saturation mobility 0.26 cm^2^V^−1^s^−1^ and good gate modulation (**e**) Perfect photoresponse with I_light_/I_dark_ = 4.06 × 10^5^ (white light, light density 3.75 mw/cm2, V = 80V). (**f**) Inverter fabricated on the PI substrate by *in-situ* growth showing a good performance.

Organic field effect transistors (OFETs) were constructed based on these well-aligned ribbons on the Si/SiO_2_ substrate in a bottom-gated top-contact configuration by manually gluing Au-films^53^,^54^ (Fig. 5a). Considering these crystals not fully covering the channel, the active channel width and length were measured from the contacting area of the crystals which crossed the drain and source electrodes. The distribution of the mobility based on 18 devices was shown in Fig. 5b. OFETs exhibit superior performance with the average mobility extracting from the saturation region of 0.2 cm^2^ V^−1^s^−1^ (the highest 0.47 cm^2^ V^−1^s^−1^ shown in Fig. S8) and on/off ratio up to 5.0 × 10^6^. The typical transfer characteristic of the device was shown in Fig. 5c, and the mobility of 0.26 cm^2^ V^−1^s^−1^ was calculated. The output characteristic was shown in Fig. 5d. The device also showed the expected gate modulation of the drain current (*I*_*D*_) in both the linear and saturation regimes. In addition, our single crystal arrays showed a very high photoswitch performance with on/off current ratio up to 4.1 × 10^5^ (Fig. 5e), which was one of the highest values reported for organic materials^47^. The single crystal arrays on the PI substrate also shared the similar electrical properties (Fig. S9). Furthermore, we fabricated an inverter which was the basic gate circuit for large scale integrated circuits on the PI substrate with patterned electrodes by *in-situ* growth (inset of Fig. 5f) and its gain was about 1.3. Besides BPEA, our method could also successfully help other materials to form single-crystal arrays with the applicable condition, such as C6-DBTDT^56^ (Fig. S10a, 1 mg/ml, 150 °C) and triisopropylsilylethynyl (TIPS)-pentacene (Fig. S10b, 0.5 mg/ml, 130 °C). The typical transfer and output characteristics of these devices were shown in Fig. S10c,e (C6-DBTDT) and Fig. S10d,f (TIPS-pentacene). Both of these arrays showed satisfying performance, which indicated our method had good universality in preparing organic single-crystal arrays.

In conclusion, we develop a new method called RASSS which takes advantage of a tiny slope and rapid annealing to fabricate desired-orientation, highly-ordered single crystal arrays of organic semiconductor BPEA. Compared with other reported methods, our process is the simplest (without any complicated procedures), the fastest (only takes a few seconds) and independent of the substrate. The average mobility is 0.2 cm^2^ V^−1^s^−1^ (the highest 0.47 cm^2^ V^−1^s^−1^) and the ratio of on/off above 10^6^. In addition, a very high photoswitch performance with on/off current ratio up to 4.1 × 10^5^ was achieved. It is believed that this method provides a new direction to fabricate single crystal arrays and the potential for the practical applications of large area organic electronics.

## Methods

### Preparation process of substrate

Si/SiO_2_, glass and polyimide were used as the substrates to fabricate the BPEA single crystal arrays. The Si/SiO_2_ substrates were cleaned in hot sulphuric acid (98%) and hydrogen peroxide (V/V = 7:3) mixed solution for 10 minutes to get rid of organic compounds and then cleaned with deionized water, acetone, and isopropyl alcohol in ultrasound for 10 minutes respectively, at last dried with N_2_. The substrates were further cleaned in oxygen plasma for 5 minutes (150 W). The glass substrates were cleaned by ultrasound in deionized water, acetone and isopropyl alcohol for 10 minutes and repeated again. The glass substrate was also cleaned by oxygen plasma for 5 minutes (150 W) before using. The polyimide substrates were prepared as follows: firstly, the indium tin oxide/glasses were cleaned by the same way with glass, and then the polyimide precursor was spin-coated on the ITO and annealed to obtain polyimide substrate based on previous reports^49^,^57^,^58^.

### Device fabrication

The single-crystal arrays on the Si/SiO_2_ and PI were used to fabricate bottom-gate, top-contact configured transistors. The gold source and drain were deposited by manually gluing Au-films. The details were to transfer the Au-film from the SiO_2_/Si substrate (with 100 nm Au) to the surface of these single crystal arrays by a probe (Keithley 4200-SCS semiconductor analyzer) in ambient atmosphere^53^,^54^. The channel length and width were measured by optical microscope. The inverter was achieved on the PI substrate, where the source and drain electrodes were patterned by photolithography. The photoswitch devices were fabricated in the same way with FETs.

### Characterization

All FETs and photoswitch devices were measured by Keithley 4200-SCS semiconductor analyzer in ambient atmosphere. The optical microscope images were taken by an OLYMPUS PX-5I-P microscope. AFM was recorded on a Nanoscope IIIa atomic force microscopy in tapping mode. The transmission electron microscopy (TEM) measurements were carried out using a JEOL-2012 instrument.

## Additional Information

**How to cite this article**: Li, Y. *et al.* Quick Fabrication of Large-area Organic Semiconductor Single Crystal Arrays with a Rapid Annealing Self-Solution-Shearing Method. *Sci. Rep.* **5**, 13195; doi: 10.1038/srep13195 (2015).

## Supplementary Material

Supplementary Information
