# Supplementary material for: Quick Fabrication of Large-area Organic Semiconductor Single Crystal Arrays with a Rapid Annealing Self-Solution-Shearing Method
Source: Sci Rep. 2015 Aug 18;5:13195. doi: 10.1038/srep13195 (PMC4539534; doi:10.1038/srep13195)
Supplement: Supplementary Information [file srep13195-s1.pdf]

Title:

Quick Fabrication of Large-area Organic Semiconductor Single Crystal Arrays with a Rapid Annealing Self-Solution-Shearing Method

Authors:
Yunze Li[1], Deyang Ji[1], Jie Liu, Yifan Yao, Xiaolong Fu, Weigang Zhu, Chunhui Xu, Huanli Dong, Jingze Li[1]*, Wenping Hu[2]*

Supporting information 1

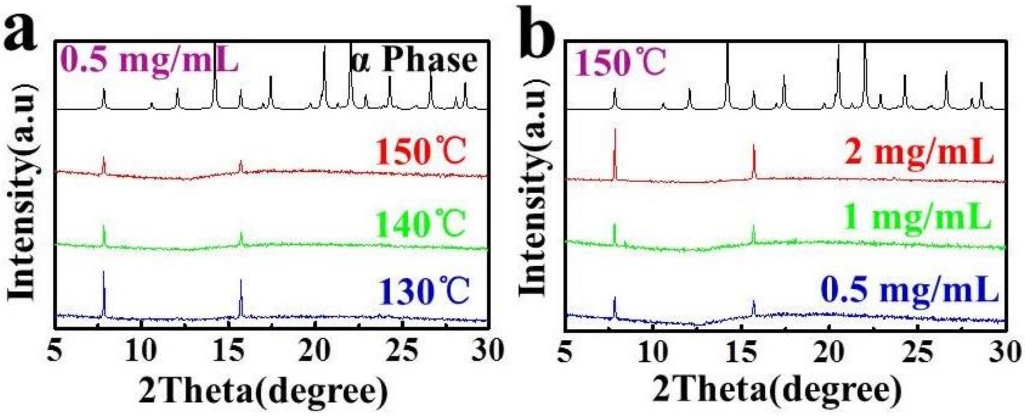

Supplementary Figure S1 | XRD characterizations at different conditions.(a)XRD patterns of the arrays obtained at the same concentration (0.5 mg/ml) but different temperatures on the SiO_2 substrate. (b)XRD patterns of the arrays obtained at the same temperature (150 ^{o}C) but different concentrations on the SiO_2 substrate. These XRD patterns show that the single crystals keep the same microstructure fabricated at different conditions.

Supporting information 2

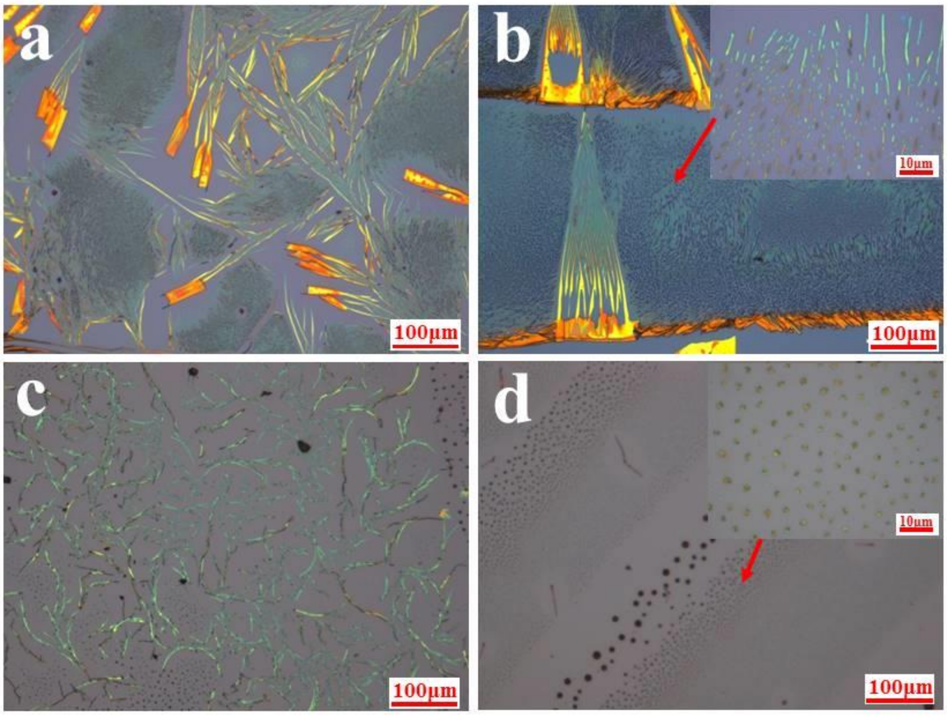

Supplementary Figure S2| Critical factors in our experiment. The effect of the substrate tilting angle and the temperature is investigated by an optical microscope. With the concentration fixed at 0.5mg/mL and the temperature fixed at 140 ^{o}C, (a) A lot of disorderly and randomly arranged crystals are observed on a horizontal substrate (0˚). (b) While the slope angel is about 1.71˚, which is formed through a piece of silicon wafer, the crystals are discontinuous featuring with low yielding. With the tilting angle fixed at 0.57˚ and the solution concentration fixed at 0.5mg/mL, (c) The phenomenon obtained at a lower temperature 120 ^{o}C, disorderly and randomly (d) Discontinuous spots are observed at a higher temperature 160 ^{o}C. It is obvious that the temperature and the slope angle have important impacts on the formation of the crystal arrays, which needs to be carefully optimized.

Supporting information 3

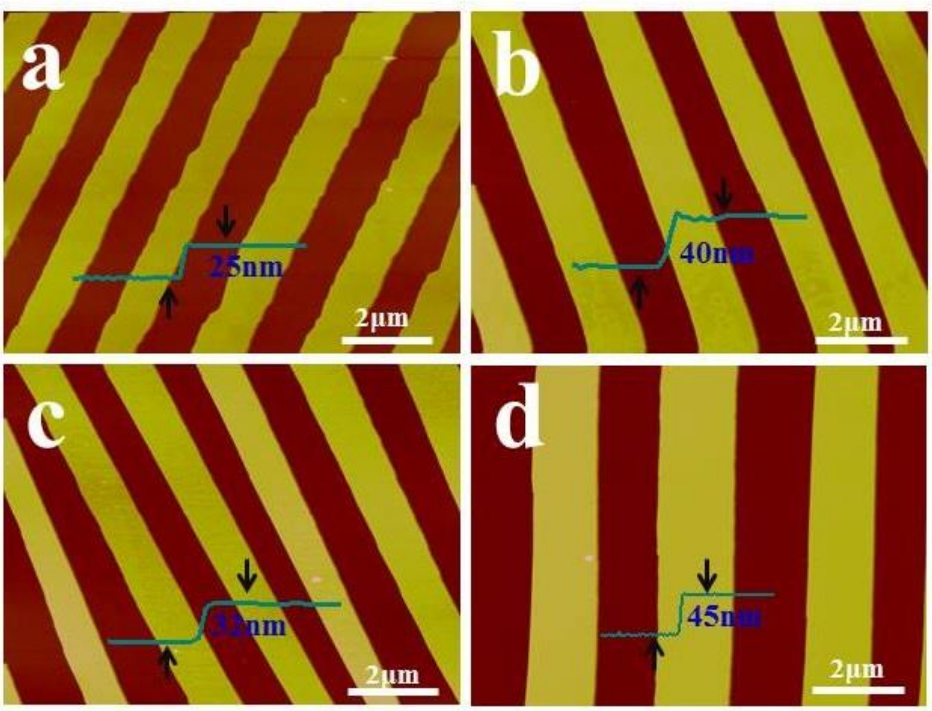

Supplementary Figure S3| AFM height mode images of the single crystals generated at the same substrate but different growth conditions. (a) 140 ^{o}C, 0.5 mg/ml. (b) 140 ^{o}C, 1mg/ml. (c) 150 ^{o}C, 0.5 m/ml. (d) 150 ^{o}C, 1mg/ml. These images illustrate that the single crystals keep smooth surfaces and regular shapes at different temperatures and concentrations, and the height of the single crystals is sensitive to the solution concentration.

Supporting information 4

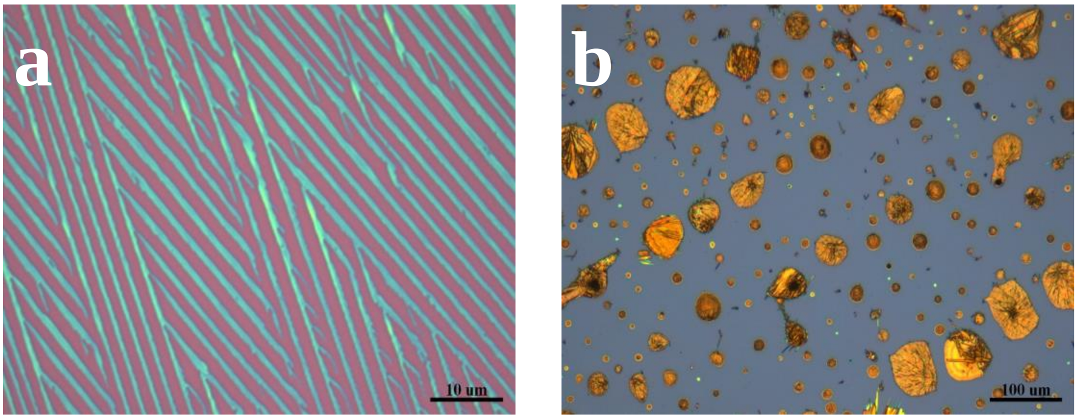

Supplementary Figure S4| The effect of solvent with different boiling points. (a) methylbenzene, (b) 1,2-dichlorobenzene.

Supporting information 5

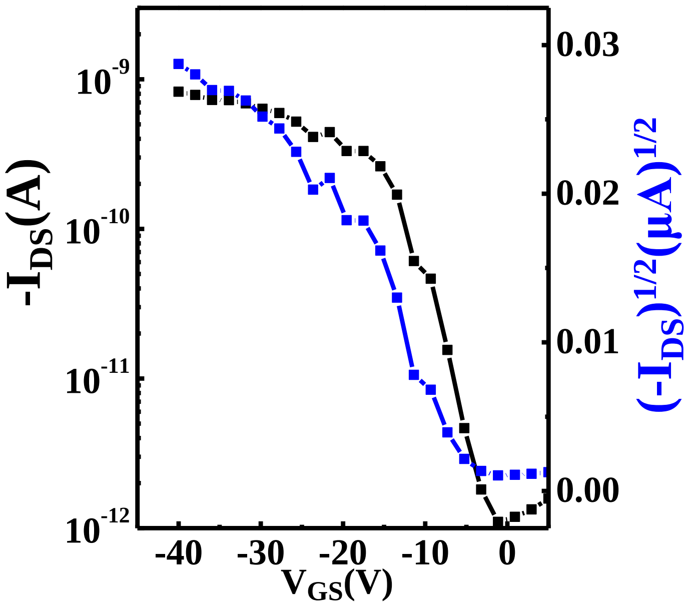

Supplementary Figure S5| The transfer characteristic of the transistor with the mobility of 2.25 x 10^{-3} cm^2 $V^{-1}s^{-1}$.

Supporting information 6

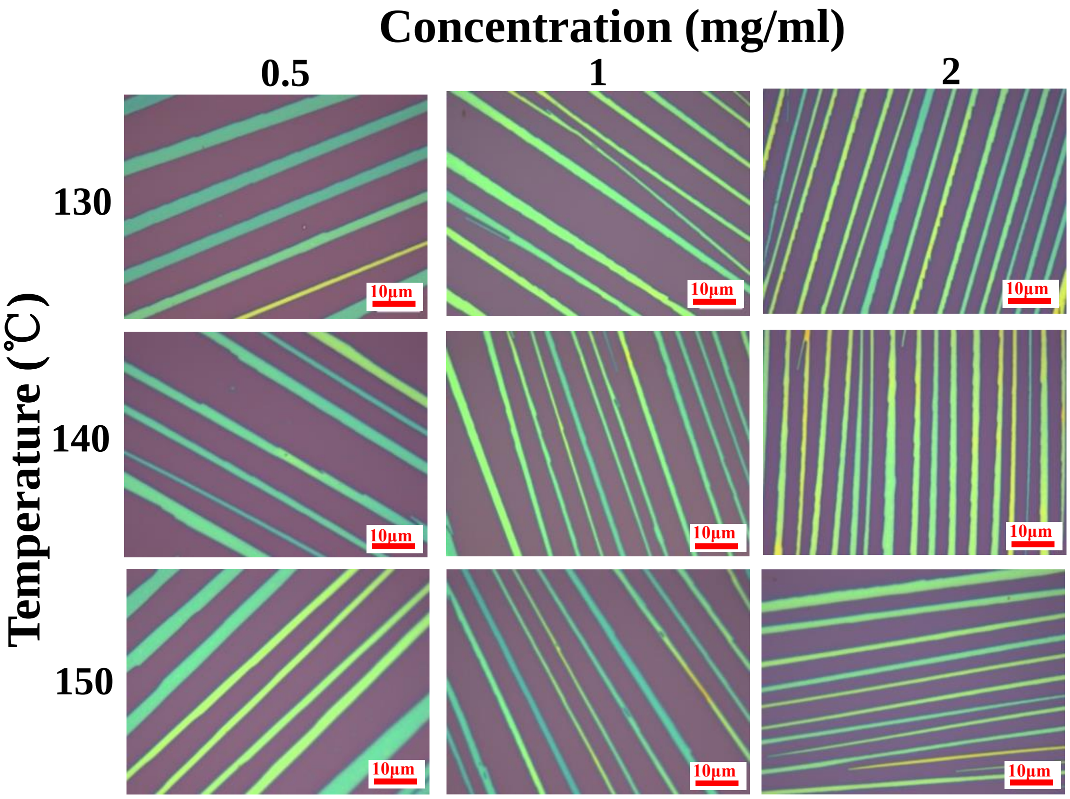

Supplementary Figure S6| Controlling the density of single crystal arrays. At the same temperature, the density increases as the concentration varies from 0.5mg/ml to 2mg/ml. However, the temperature has no visible effect on the density while the concentration is fixed. Thus, we can control the density of the array by modulating the solution concentration.

Supporting information 7

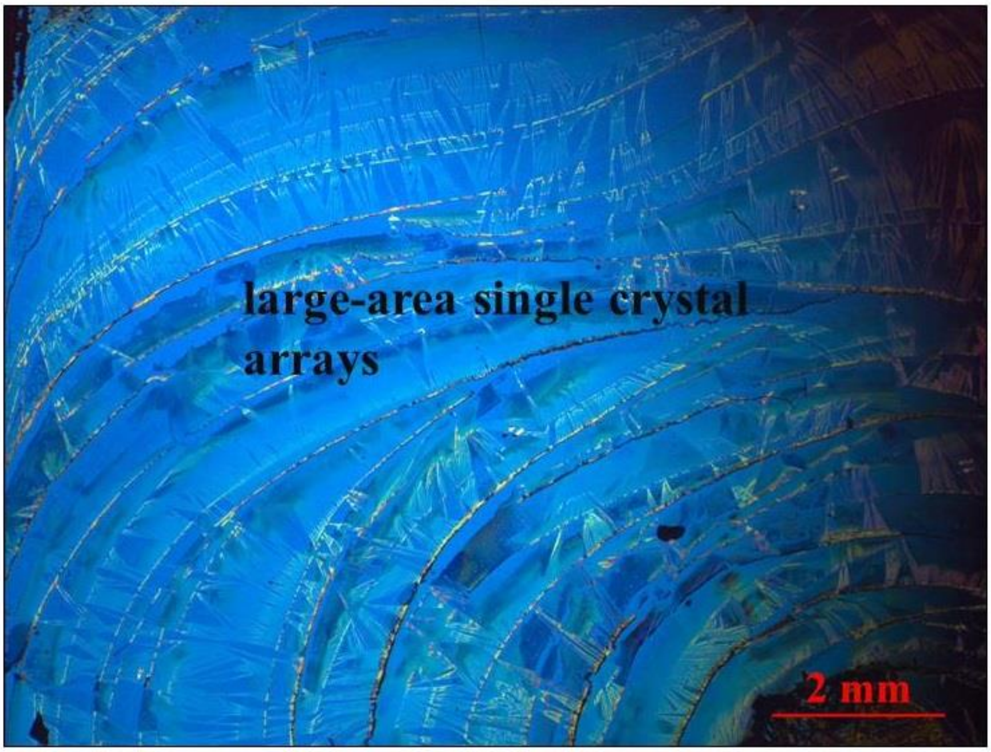

Supplementary Figure S7| Optical microscope image collected from the whole substrate. It is clear that the BPEA single crystal ribbon arrays can be achieved on the whole substrate, where each experiment can fabricate the large area in the square centimeter scale within 10 s.

Supporting information 8

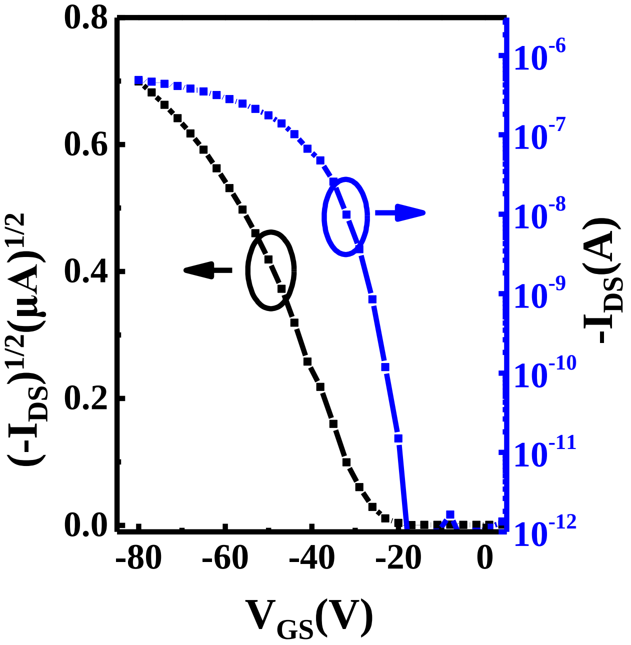

Supplementary Figure S8| The transfer characteristic of the transistor with the highest mobility of 0.47 $cm^2\,V^{-1}s^{-1}$.

Supporting information 9

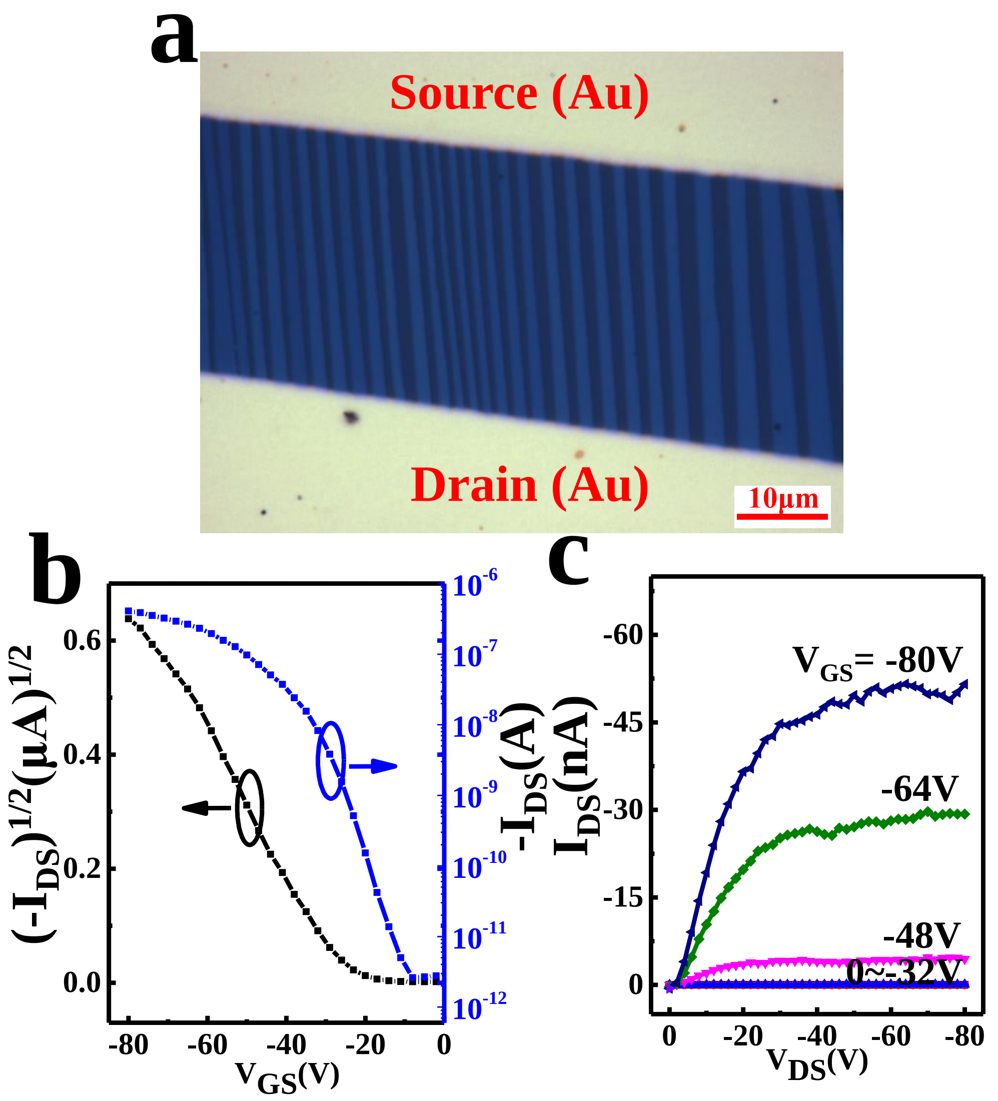

Supplementary Figure S9| Organic field effect transistor fabricated on the PI substrate.(a) Optical microscope image of the FET. (b) Transfer (c) Output characteristics of a typical device.

Supporting information 10

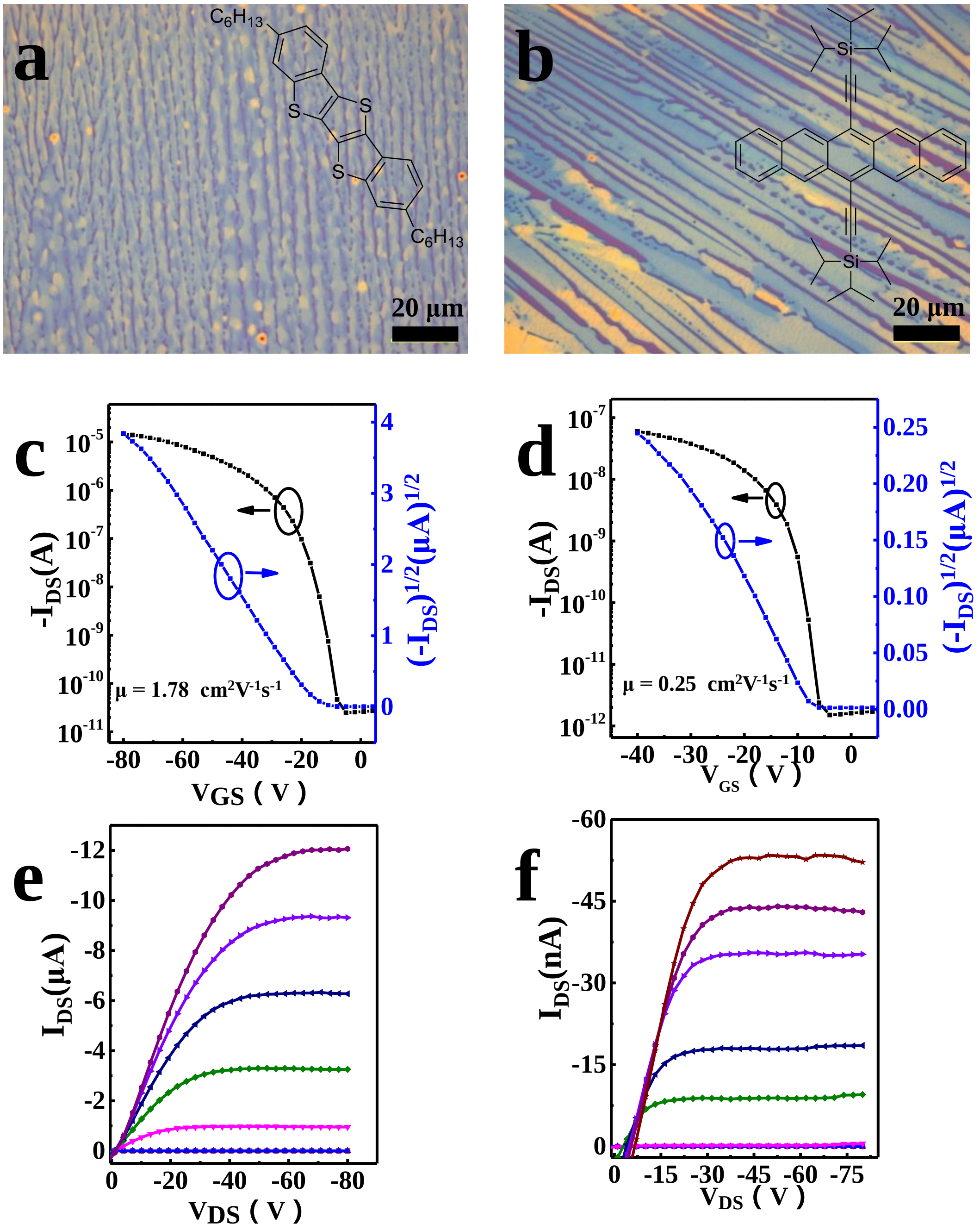

Supplementary Figure S10| Organic field effect transistor fabricated using C6-DBTDT and TIPS-pentacene. (a) Optical microscope image of the C6-DBTDT arrays, (b) Optical microscope image of the TIPS-pentacene arrays, Transfer (c) and Output (e) characteristics of the device based on C6-DBTDT arrays, Transfer (d) and Output (f) characteristics of the device based on TIPS-pentacene arrays.
